# Functionalization of Petroleum Coke-Derived Carbon for Synergistically Enhanced Capacitive Performance

**DOI:** 10.1186/s11671-016-1382-0

**Published:** 2016-03-24

**Authors:** Yan Zhang, Xuejin Li, Jufeng Huang, Wei Xing, Zifeng Yan

**Affiliations:** School of Chemical Engineering, State Key Laboratory of Heavy Oil Processing, China University of Petroleum, Qingdao, 266580 People’s Republic of China; School of Science, State Key Laboratory of Heavy Oil Processing, China University of Petroleum, Qingdao, 266580 People’s Republic of China

**Keywords:** Petroleum coke, Activated carbon, Oxygen doping, Supercapacitor, Pseudo-capacitance

## Abstract

Petroleum coke is a valuable and potential source for clean energy storage if it could be modified legitimately and facilely. In the present study, porous carbon with high surface area and abundant oxygen-containing groups was prepared from petroleum coke by chemical activation and modification processes. The as-prepared carbon exhibits a high surface area (1129 m^2^ · g^−1^) and stable micrographic structure. It presents a high specific capacitance and excellent rate performance in KOH electrolyte. Even at an ultrahigh current density of 50 A · g^−1^, the specific capacitance of the prepared carbon can still reach up to an unprecedented value of 261 F · g^−1^ with a superhigh retention rate of 81 %. In addition, the energy density of this material in aqueous electrolyte can be as high as 13.9 Wh · kg^−1^. The high energy density and excellent rate performance ensure its prosperous application in high-power energy storage system.

## Background

Environment-friendly energy supply is seemed to be one of the biggest concerns right now, which is closely associated with our lives [1]. It is undeniable that fossil energy will continue to play a major role in meeting our energy requirements for an extended period. However, each kilowatt hour of electricity generated by burning coal coproduces an average 1000 g CO_2_ emission [2], which will cause global warming. In addition, portable electronic devices and hybrid electric vehicles, with power source of electric energy resource, are growing very fast in recent years. In this regard, efficient electrical energy storage systems have gradually caused extensive concern [1, 3–5]. Supercapacitors are very attractive owing to their superior power density (>10 kW · kg^−1^), fast charge/discharge rates (within seconds), and long cycle lifetime (>10^5^) compared to other chemical energy storage devices [6, 7]. They can play a vital role in some applications that needed high-power delivery while batteries cannot meet the requirement.

Supercapacitors can be divided into two categories according to the charge storage mechanism [8]. One is electrical double-layer capacitors (EDLCs) which store energy by the adsorption of both anions and cations. The other category is the pseudo-capacitors that store energy through fast surface redox reactions. Carbon-based materials have been widely investigated as electrodes of supercapacitors due to their desirable physical and chemical properties [9–12]. These properties include low cost, ease in processing, controllable porosity, and electrocatalytic active sites for a variety of redox reactions. Large accessible specific surface area and appropriate pore size of the carbon-based electrodes are crucial to ensure a good performance of EDLCs in terms of both power delivery rate and energy storage capacity. Activated carbons (ACs) are often considered as EDLC electrode materials because of their high surface area and somewhat controllable pore size. For example, a mesoporous activated carbon sphere derived from resorcinol-formaldehyde resin has been prepared and chosen as electrode of EDLC [13]. It possesses a high surface area and presents a good electrochemical double-layer capacitive performance. Carbon precursors like such resin are too numerous to mention [14–21]. Among them, petroleum coke (PC) is a better candidate for preparing high surface area AC due to its high carbon content and low ash content. In addition, it is abundant and cheap (less than $100 per ton and usually used as burning fuel) in China. Lee and Choi [20] prepared a high surface area activated carbon derived from high-sulfur petroleum cokes by chemical activation using KOH. Its surface area can reach up to 1980 m^2^g^−1^ with a high KOH to coke ratio of 4. The surface area can even reach up to as high as 3000 m^2^g^−1^ when the KOH to coke ratio increases to 10 [21]. It is obvious that PC is very difficult to be activated due to its stable micrographic structure and lack of the initial pores [22]. During the activation of PC, large quantity of KOH was expended to obtain high surface area AC, which was uneconomic and eco-unfriendly. In addition, pure electrochemical double layer (EDL) capacitance from AC is relatively low, resulting in an inferior energy density.

Heteroatom doping seems to be an effective way to solve these problems. It can not only make PC easy to be activated [23] but also introduce pseudo-capacitance to enhance the overall capacitance of the electrode materials. Lu et al. [24] studied the oxidization and activation mechanism of PC and found that the oxygen-containing functional groups play an important role during the activation process. Bai et al. [18] prepared a nitrogen-doped AC from PC by using urea and KOH as activating agent. The as-prepared AC possesses a high level of nitrogen and large surface area, leading to an improved CO_2_ uptake capacity. Jiang et al. [25] synthesized ACs from PC by the combination of chemical treatment with HClO_4_ or H_2_O_2_ and chemical activation with KOH. The resulting activated carbon had higher specific surface area and better iodine adsorption value. Unfortunately, the influence of functionalization of PC-derived carbon on its energy storage performance has rarely been studied, limiting its wide use in clean energy storage applications.

Therefore, the purpose of this research is to investigate the effects of oxygen doping of petroleum coke-derived carbon (PCAC) on its capacitive performance. Different oxidants, such as H_2_O_2_ or HNO_3_, were used to tune the surface chemistry property of PCAC. It is found that oxidization by H_2_O_2_ can effectively influence the pore structure and capacitive performance of PCAC. The introduced functional groups not only enhance the EDL capacitance but also provide pseudo-capacitance, leading to an enhanced overall capacitance.

## Methods

### Materials

A typical Chinese PC from Shengli refinery was used as a raw material. It was ground and sieved to select the grains in the range of 100–149 μm. The obtained powder was used as precursors of active carbon after drying in an oven at 383 K for 3 h. KOH, H_2_O_2_, HNO_3_, and HCl of analytical grade were obtained from Sinopharm Chemical Reagent Co., Ltd. and used without further purification.

### Synthesis of Surface-modified Activated Carbon Derived from PC

The samples were prepared from PC with a combination of KOH activation and chemical modification using hydrogen peroxide or nitric acid as oxidizing agent.

#### KOH Activation

At a KOH/PC mass ratio of 2:1, 10.0 g PC was mixed with KOH. The mixture was transferred into a crucible and carbonized at 850 °C for 2 h with a heating ramp of 5 °C min^−1^ in argon. The carbonized sample was washed and filtered with HCl aqueous solution and distilled water successively until the pH of the filtrate was 7. After rinsing, the sample was dried overnight at 120 °C and named as PCAC.

#### Chemical Modification

In a typical procedure, 3.0 g PCAC power was first added into 30 mL 20 % H_2_O_2_ or 1 M HNO_3_ solution, then the solution was transferred to autoclave and stirred for 8 h at 353 K. After that, the sample was thoroughly washed with distilled water to remove excess H_2_O_2_ or HNO_3_. The obtained materials were dried at 373 K in an oven for 12 h. The samples via modification with H_2_O_2_ and HNO_3_ are denoted as PCHO and PCNC, respectively.

### Material Characterization

The as-prepared carbons were characterized by Raman spectroscopy (JY Labram HR 800, HORIBA Jobin Yvon, *λ* = 514 nm) for structure detection. The surface chemical properties of the samples were characterized by Fourier transform infrared spectroscopy (FT-IR) (Nicolet 6700, Thermoscientific). An elemental analyzer (ANTEK, ANTEK 9000, USA) was used to analyze the elemental content of the synthesized samples. The microscopic morphologies of the samples were observed using scanning electron microscope (SEM, S-4800 Hitachi) and transmission electron microscopy (TEM, JEM-2100 JEOL) techniques. Nitrogen adsorption-desorption measurements were performed on a Tristar 3000 analyzer (Micromeritics, USA) to obtain specific surface area and pore structure parameters of the samples. The specific surface area of the samples was calculated by the Brunauer-Emmett-Teller (BET) method in the relative pressure (*P*/*P*_0_) between 0.06 and 0.3, and the total pore volume was determined from the amount of nitrogen uptake at a relative pressure (*P*/*P*_0_) of 0.99.

### Electrochemical Measurement

The electrochemical capacitive performance measurements were carried out by a CHI 660D instrument using a three-electrode system. The three-electrode system includes platinum film as the counter electrode, saturated calomel electrode (SCE) as the reference electrode, and the as-prepared sample as the working electrode. The carbon materials were mixed with polytetrafluoroethylene as a binder at a weight ratio of 95:5. After drying at 80 °C overnight, the mixture was smeared on a nickel foam and pressed at 100 kg/cm^2^ to form a slice as a working electrode. The loading amount of active materials for a single electrode was 2 mg. The electrochemical performances of the carbon materials were measured in a 30 wt% KOH solution. A potential window of −1~0 V vs. SCE was selected for the electrochemical measurements. The average specific capacitance (*C*) was derived from the discharge curve according to the following equation:

$$ \mathrm{C}=\frac{I\varDelta t}{m\varDelta V} $$

From the discharge curve measured with the two-electrode system, the energy density (*E*) and power density (*P*) were calculated as follows [26]:

$$ \mathrm{E}=I{\displaystyle \int}\frac{Vdt}{2m} $$

$$ \mathrm{P}=\frac{E}{\varDelta t} $$

where *C* is the specific capacitance (*F* · *g*^−1^), *I* is the discharge current (*A*), ∆*t* is the discharge time (s), ∆*V* is the potential change during discharge process (*V*), and *m* is the mass of active material in a single electrode (g).

## Results and Discussion

### Morphological Evolution

**Fig. 1:**
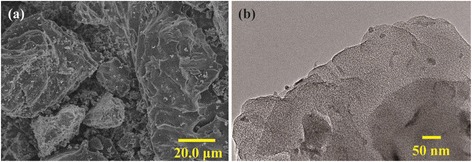
**a** SEM and **b** TEM images of PCAC

The morphology and microstructure of PCAC was investigated by SEM and TEM. As shown in Fig. 1a, PCAC has relative coarse morphology and evident fracture. The TEM image (Fig. 1b) of PCAC clearly illustrates the amorphous, worm-like micropores formed during the activation process. The irregular particles with abundant micropores can provide a large adsorption interface for electrolyte ions, which is beneficial for its EDL capacitive performance.

### Textural and Surface Properties of Samples

**Fig. 2:**
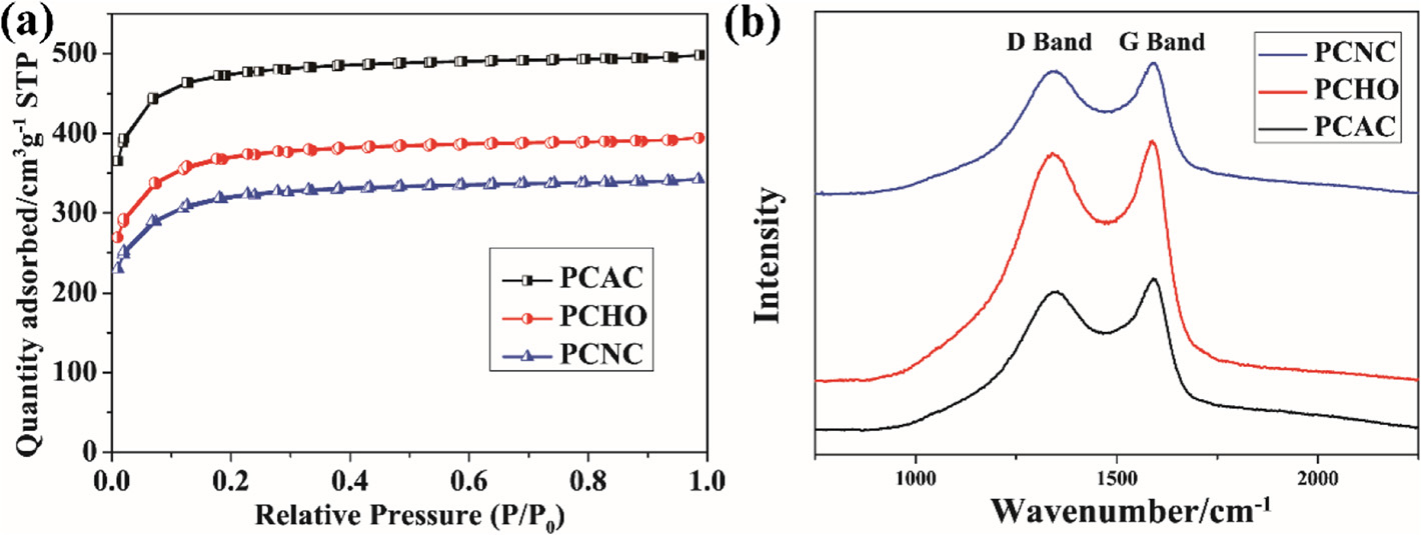
**a** Nitrogen adsorption-desorption isotherms and **b** Raman spectra of PCAC, PCHO, and PCNC

The BET specific surface area and pore-structure parameters of the as-prepared carbons were determined by N_2_ adsorption-desorption analyses. N_2_ adsorption-desorption isotherms (Fig. 2a) of all carbon samples are typical type-I (IUPAC), which is a characterization of the micropore [23]. However, in comparison with petroleum coke derived activated carbon (PCAC), H_2_O_2_ modified PCAC (PCHO), and HNO_3_ modified PCAC (PCNC) showed smaller N_2_ adsorption capabilities. As listed in Table 1, PCAC possesses the highest specific surface area of 1441 m^2^ · g^−1^ and total volume of 0.77 cm^3^ · g^−1^. It is obvious that most of the surface area originates from micropores which is important to the EDL capacitance of PCAC due to the distortion of solvent shell of ions in micropores and the decrease in thickness of EDL [27]. After chemical modification by H_2_O_2_ or HNO_3_, the specific surface areas of PCHO and PCNC decreased to 1129 and 978 m^2^ · g^−1^, respectively. The micropore surface areas of both PCHO and PCNC decreased a lot, whereas their mesopore surface areas increased. This is because of the destruction of pore walls and micropore blocking by oxygen-containing groups introduced during the chemical modification [28, 29].

**Table 1:** Textural properties of the carbon materials

Samples	*S*_BET_^a^ [m^2^/g]	*S*_Micro_^b^ [m^2^/g]	*S*_Meso_^c^ [m^2^/g]	*V*_Total_^d^ [cm^3^/g]	*V*_Micro_^b^ [cm^3^/g]	*V*_Meso_^c^ [cm^3^/g]	*D* ^e^ [nm]
PCAC	1441	1192	190	0.77	0.62	0.12	2.1
PCHO	1129	836	211	0.61	0.43	0.13	2.2
PCNC	978	705	196	0.53	0.37	0.12	2.2

^a^BET (Brunauer-Emmett-Teller) surface area

^b^The micropore surface area *S*_Micro_ and micropore volume *V*_Micro_ were calculated from the *t* plot method

^c^The mesopore surface area (*S*_Meso_) and mesopore volume (*V*_meso_) were calculated from the BJH (Barrett-Joyner-Halenda) method

^d^Total pore volume, measured at *P*/*P*_0_ = 0.99

^e^Average pore size, calculated by 4 *V*_Total_/*S*_BET_

Raman spectroscopy is an effective tool to distinguish ordered and disordered skeletal structure of carbon. The Raman spectra of all samples (Fig. 2b) revealed two bands at ~1350 and ~1590 cm^−1^ corresponding to the D and G bands of carbon, respectively. The D band reflects the defects and disorder in the samples, while the G band represents the ordered sp^2^-carbon plane [30, 31]. The intensity ratio of the D and G band (*I*_D_/*I*_G_) was used to evaluate the disorder degree and average size of the sp^2^ domains of the materials. The calculated *I*_D_/*I*_G_ values are listed in Table 2. The *I*_D_/*I*_G_ of PCAC, PCHO, and PCNC are 0.95, 0.94, and 0.91, respectively. After oxidization process, both the *I*_D_/*I*_G_ values of PCHO and PCNC decreased slightly, indicating that the oxygen-containing groups mainly anchored on the defects of the PCAC and the sp^2^ carbon plane was not damaged. In addition, the size of graphitic carbon (La) was calculated according to the empirical equation La = 4.4 *I*_G_/*I*_D_ (nm) [32]. La of PCAC, PCHO, and PCNC are 4.6, 4.7, and 4.8 nm, respectively. These results illustrate that the petroleum coke-derived carbons possess practically graphitized microstructure which is beneficial to fast electron transfer.

**Table 2:** The intensity ratios of *I*_D_/*I*_G_ in Raman spectra of carbon samples and the size of their graphitic carbon

Samples	*I*_D_/I_G_^a^	La (nm)
PCAC	0.91	4.6
PCHO	0.94	4.7
PCNC	0.95	4.8

*La* the size of graphitic carbon

^a^ *I*_D_/*I*_G_ was calculated by the intensity of D band and G band

**Fig. 3:**
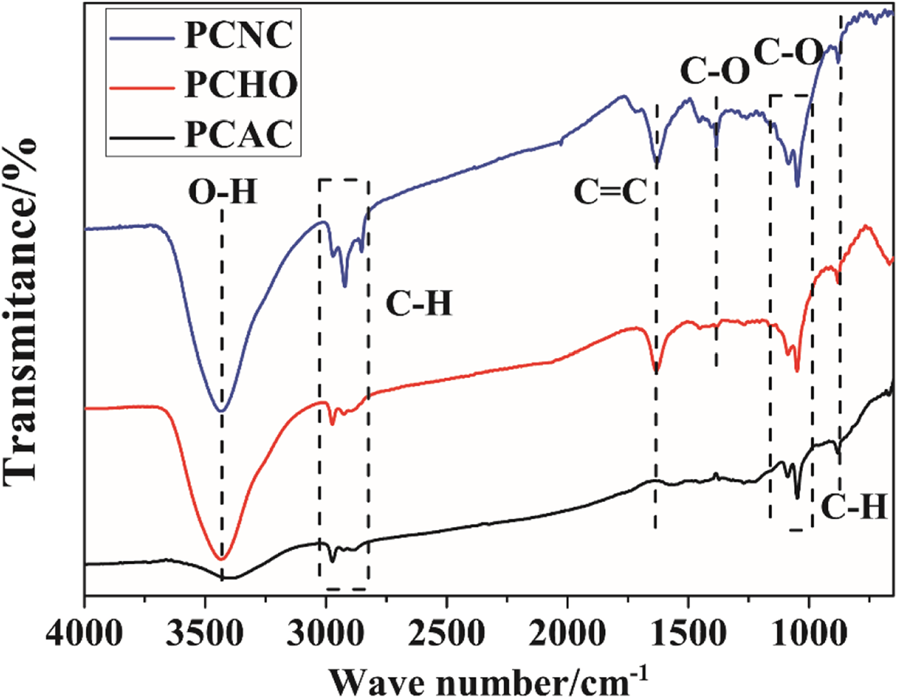
FT-IR spectra of PCAC, PCHO, and PCNC

The FT-IR spectra of the samples are shown in Fig. 3. All samples exhibit a series of IR bands. The broad band at about 3433 cm^−1^ was associated with the –O–H stretching vibration mode of hydroxyl functional groups [25]. The bands appeared at 1636 cm^−1^ are assigned to the C=C aromatic bonds [33]. The alkoxy group can be confirmed by the C–O band around 1046 cm^−1^. It is worth noting that a new band at around 1386 cm^−1^ was observed in the spectra of PCHO and PCNC. This band corresponds to the bending of tertiary C-OH groups introduced by H_2_O_2_ or HNO_3_ during the chemical modification [33]. In addition, compared with PCAC, both PCHO and PCNC present a much more intense hydroxyl adsorption band, also indicating the effective oxidization of H_2_O_2_ and HNO_3_. The element composition of these samples is listed in Table 3. It can be seen that the oxygen content of PCHO is the highest, while that of PCAC is the lowest. The elemental analysis results also confirm the effective oxidization of H_2_O_2_.

**Table 3:** The elemental composition of samples

Samples	Element content (%)		
	C%	O%	N%
PCAC	91.75	6.66	1.22
PCHO	78.05	19.09	1.13
PCNC	83.93	13.95	1.45

### Electrochemical Capacitive Performance

**Fig. 4:**
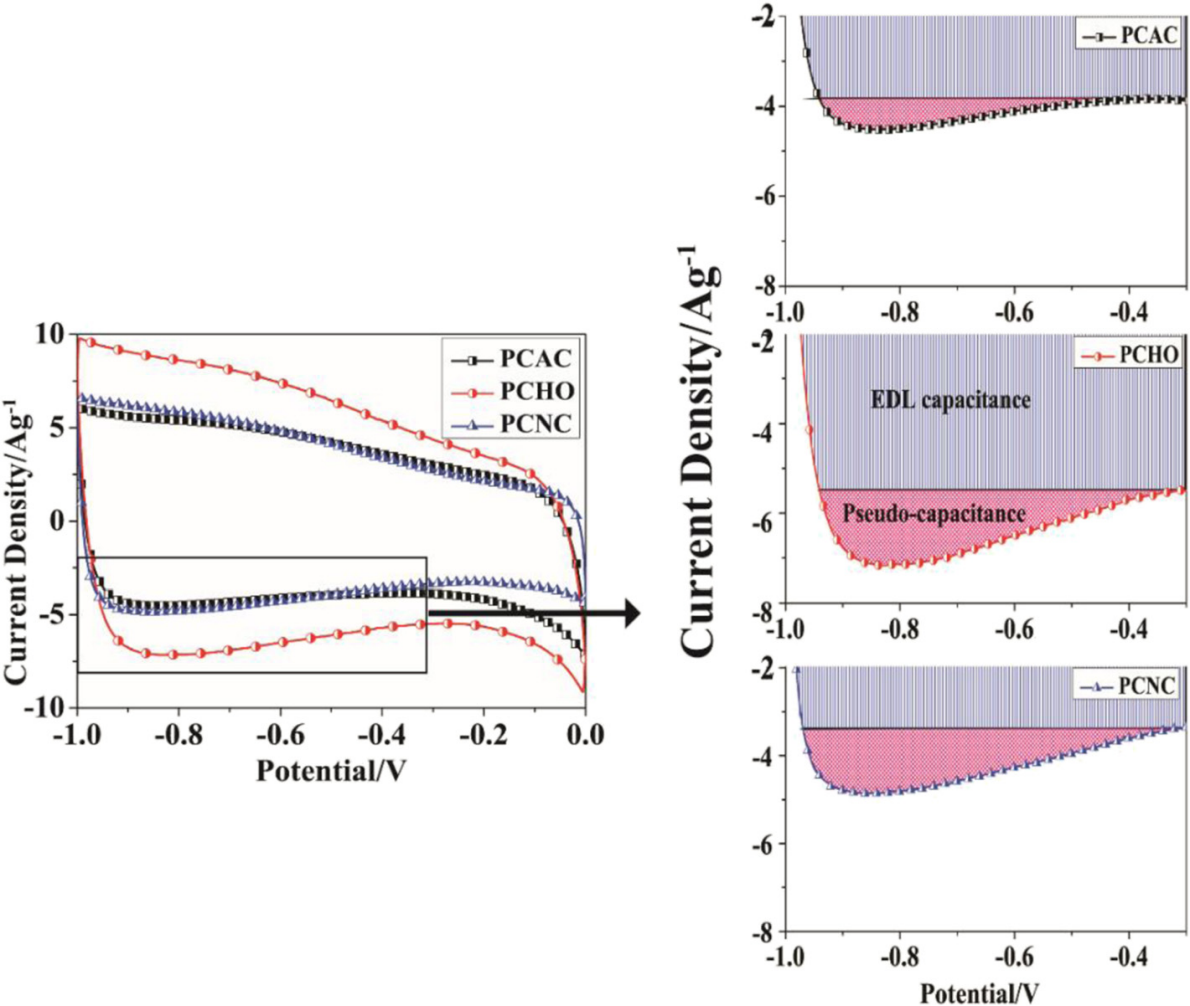
Cyclic voltammograms of PCAC, PCHO, and PCNC at a scan rate of 20 mVs^−1^

The electrochemical performance of PCAC, PCHO, and PCNC were firstly investigated using a three-electrode system in the electrolyte of 30 wt% KOH solution. Cyclic voltammograms of the as-prepared samples at a scan rate of 20 mV · s^−1^ are shown in Fig. 4. The curves of samples display an irregular rectangle-like shape with a wide hump in the voltage range of −1.0~−0.3 V, suggesting that the charges are stored via both ion adsorption and faradic redox reaction. It is important to note that the integrated area with the CV curve of PCHO is the largest, suggesting that the highest specific capacitance was achieved for PCHO. As illustrated in Fig. 4, PCHO possesses both higher EDL capacitance and pseudo-capacitance than PCAC and PCNC. Although the specific surface area of PCHO is lower than that of PCAC, PCHO presents a much larger EDL capacitance than others. This is because that the functional groups usually block the ultramicropores of the material which are usually inaccessible for electrolyte ions at relative high current density. In addition, the abundant oxygen-containing functional groups in PCHO can enhance the accessibility of electrolyte, leading to a more sufficient utilization of the micropore surface area [34]. In addition, the plentiful oxygen-containing groups can provide large pseudo-capacitance, thus enhancing the overall capacitance of PCHO. Compared with PCAC, PCNC displays a lower EDL capacitance and a higher pseudo-capacitance, resulting in a similar specific capacitance with PCAC. The lower EDL capacitance is caused by the less interface for ion adsorption, as PCNC present much less surface area than PCAC. The CV results reveal that the oxidization of PCAC with H_2_O_2_ is an effective way to introduce considerable pseudo-capacitance and enhance the EDL capacitance.

**Fig. 5:**
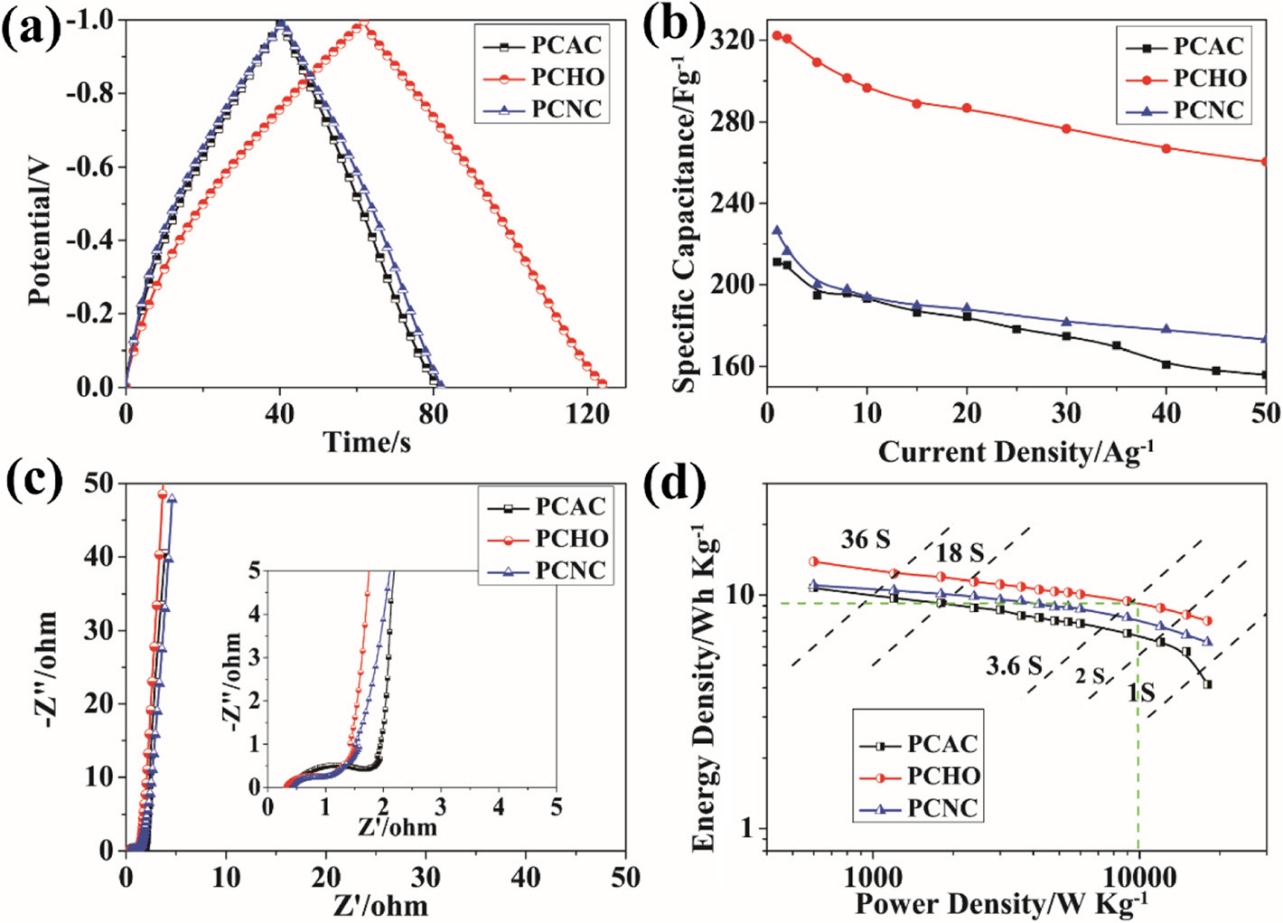
**a** Galvanostatic charge-discharge curves of PCAC, PCHO, and PCNC at a current density of 5 A · g^−1^ in 30 wt% KOH. **b** Variation in the specific capacitance of PCAC, PCHO, and PCNC as a function of current density. **c** Nyquist plots. **d** Ragone plots of the PCAC, PCHO, and PCNC

The galvanostatic charge-discharge (GCD) curves at 5 A · g^−1^ of the samples are displayed in Fig. 5a. It is obvious that the curves of all samples exhibit a slight distorted triangular shape, which is caused by the pseudo-capacitive behavior of the oxygen-containing functional groups. The discharge time of PCHO is much longer than those of PCAC and PCNC, indicating its largest specific capacitance. A plot of the specific capacitance versus current density (Fig. 5b) was further collected to study the rate performance. It is found that PCHO shows the largest specific capacitance at all current densities. The maximum specific capacitance of 322 F · g^−1^ was attained at a current density of 1 A · g^−1^ for PCHO, which is much higher than those of PCAC (211 F · g^−1^) and PCNC (226 F · g^−1^). Remarkably, the specific capacitance of PCHO can still be maintained to an unprecedented value of 261 F · g^−1^ even at the current density of 50 A · g^−1^. The retention ratio of PCHO from 1 to 50 A · g^−1^ is 81 %, which is higher than those of PCAC (76 %) and PCNC (73 %). These above results confirm that the increased amount of mesopores and introduction of abundant oxygen-containing groups are effective in improving the electrolyte accessibility, leading to fast ion response and superior rate performance of PCHO.

Electrochemical impedance spectroscopy was further used to analyze the charge transfer and ion transport of the materials. Figure 5c shows Nyquist plots in the frequency range from 100 kHz to 0.01 Hz. The near vertical line over the low-frequency ranges suggests excellent capacitive behavior. It is well known that high slope value means the fast ion transport [35]. Obviously, PCHO presents a bigger slope than PCAC and PCNC. In addition, PCHO also shows a smaller semicircle than PCAC at high-frequency region of the Nyquist plot. This is because that the higher degree of graphitization of PCHO, as illustrated by Raman results, is in favor of fast electron transfer. The energy density and power density at an operating voltage of 1.2 V for PCAC-, PCHO-, and PCNC-based ECs are plotted in Fig. 5d. It can be found that the energy density and power density of PCHO are much higher than those of PCAC and PCNC. At a very high power density of 10 kW · kg^−1^, PCHO can still deliver a high energy density of 9.2 Wh · kg^−1^, suggesting the promising application of PCHO in some scenarios requiring both high power and high energy density.

The high specific capacitance and excellent rate performance could be attributed to the following reasons: (i) The high surface area provides a large adsorption interface for electrolyte to form the EDL, leading to a great electrochemical double layer capacitance. (ii) The introduction of oxygen-containing groups can improve the wettability of PCHO in aqueous electrolyte. They can not only increase the accessibility of electrolyte ions to make full use of the surface area but also provide considerable pseudo-capacitance to enhance the overall capacitance. (iii) The stable micrographitic structure cannot be damaged during the chemical activation by moderate amount of KOH. Thus, the ordered sp^2^ carbon domains can enhance the transfer of electric charges, which reduces the internal resistance and also helps to maintain high rate performance of the supercapacitor.

## Conclusions

To promote the application of petroleum coke in clean energy storage, we have synthesized an oxygen-doped porous carbon via KOH activation and chemical modification of petroleum coke. The as-prepared carbon possesses a high surface area and abundant oxygen-containing groups, leading to large EDL capacitance and pseudo-capacitance. The introduced oxygen-containing groups can also improve the accessibility of electrolyte ions; thus, the ions can get to the surface easily even at ultrahigh current density. In addition, the stable micrographic structure of the material makes the electron transfer fast. Furthermore, the facile and efficient synthesis process could be easily scaled up, which makes the practical application of petroleum coke in energy storage possible.
